# Diabetes severely affects attentional performance after coronary artery bypass grafting

**DOI:** 10.1186/1749-8090-7-115

**Published:** 2012-11-06

**Authors:** Jens-Holger Krannich, Therese Tobias, Jens Broscheit, Rainer Leyh, Wolfgang Müllges

**Affiliations:** 1 Department of Cardiothoracic Surgery, University Hospital Würzburg, Oberdürrbacherstraße 6, Würzburg, 97080, Germany; 2 Department of Anesthesiology, University Hospital Würzburg, Oberdürrbacherstraße 6, Würzburg, 97080, Germany; 3 Department of Neurology and Comprehensive Heart Failure Center, University Hospital Würzburg, Josef-Schneider-Straße 12, Würzburg, 97080, Germany

**Keywords:** Diabetes, Coronary artery bypass graft, Cognitive, Attentional performance

## Abstract

**Background:**

Diabetes is a risk factor for (micro) vascular damage of the brain, too. Therefore cognitive performance after coronary artery bypass grafting may be hypothesized worse in diabetics. To avoid observational errors a reliable tool for testing attentional performance was used. We evaluated whether diabetes mellitus disposes to distinct cognitive dysfunction after coronary artery bypass grafting (CABG).

**Methods:**

Three aspects in attentional performance were prospectively tested with three different tests (alertness: composed of un-cued and cued reaction, divided attention, and selective attention) by a computerized tool one day before and seven days after CABG in a highly selected cohort of 30 males, 10 of whom had diabetes. Statistical comparisons were done with analysis of variance for repeated measurements and Fisher's LSD.

**Results:**

Prior to CABG there was no statistically meaningful difference between diabetics and non-diabetics. Postoperatively, diabetic patients performed significantly worse than non-diabetics in tests for un-cued (p=0.01) and cued alertness (p=0.03). Test performance in divided attention was worse after CABG but independent of diabetes status. Selective attention was neither affected by diabetes status nor by CABG itself.

**Conclusions:**

Diabetes may have an impact on cognitive performance after CABG. More severe deficits in alertness may point to underlying microvascular disease.

## Background

Postoperative cognitive dysfunction is a disturbing complication of CABG surgery. Risk factors include patient age, arterial hypertension, use of cardiopulmonary bypass [1,2]. There are conflicting data [2] on whether the uniformly detected initial postop decline is truly transient and whether it may subside if vascular risk factors are controlled [3], and which factors carry the risk of a progressive long-term decline [4]. Diabetes mellitus has been proposed as one such risk factor for a more marked postoperative impairment [1]. This seems plausible as patients with metabolic syndrome may often develop vascular encephalopathy due to progressive small vessel disease of the brain [5]. In large cohorts of post-CABG patients, attention, concentration, psychomotor speed, and not memory revealed to be most severely affected [2]. This test pattern is typically found in vascular dementia, too [6]. We hypothesized that diabetic CABG patients are prone to experience a more severe postoperative cognitive dysfunction and that this may be discovered by testing alertness and attentional deficits, as attention is the basis of a good performance of the whole cognitive system [7].

## Methods

The study was approved by the local Ethics Committee. All patients signed a written informed consent. Prospectively, serial CABG patients underwent a one day preoperative and one week postoperative testing. Inclusion criteria for this study were male sex, age younger than 80 years, native German speaker, a history of hypercholesterinemia and arterial hypertension (also accepted if values were within the normal range under appropriate treatment). Exclusion criteria were unstable angina, urgent surgery, preoperative focal neurologic signs suggesting a previous stroke, obvious dementia, or no consent. Post-surgery exclusion criteria were on-pump-CABG with an extracorporeal circulation time not between 60 to 180 minutes, sedatives at testing, a new stroke as demonstrated by repeat neurologic exam, and a subnormal glucose concentration (< 40 mg%) or diabetic excess (> 250 mg%) during the perioperative period. Additionally, the potentially confounding variables depression and anxiety were controlled by the Hospital Anxiety and Depression Scale (HADS) [8] considering a score ≥ 8 as the criterion depression or anxiety [9]. The post-op HADS evaluation was refused by six patients. For a depiction of the study population see Table 1.

**Table 1:** Patient characteristics

**Characteristic**	**Mean (SD) or number (%)**
age [years]	66,0 (7,7)
History of myocardial infarction	13/30 (43%)
3 vessel disease	30/30 (100%)
extracorporeal circulation time [minutes]	112,8 (29,6)
aortic clamping time [minutes]	68,6 (20,5)
diabetes mellitus	10/30 (33%)
ASA 3	18/30 (60%)
ASA 4	12/30 (40%)
Hypertension (controlled by drugs)	30/30 (100%)
ASS	30/30 (100%)
Statins	30/30 (100%)
sedativa at testing	0/30 (0%)
BMI > 25 [kg/m^2^]	4/30 (13%)
HADS > 8 score in depression one day pre-CABG	6/30 (20%)
HADS > 8 score in anxiety one day pre- CABG	10/30 (33%)
HADS > 8 score in depression seven days after CABG	6/24* (25%)
HADS > 8 score in anxiety seven days after CABG	5/24* (21%)

*- six patients denied to fill in HADS after CABG.

By that, thirty patients were evaluated, twenty of whom were non-diabetics (HbA1c in the normal range), and ten had a history of type 2-diabetes, all treated effectively (HbA1c < 7%) at surgery.

A computerized psychometric test device was used (Test of Attentional Performance, TAP; Psytest, Herzogenrath, Germany) validated in German speaking people (available for six languages). It was developed to identify patients with neurocognitive impairment in general [7,10,11]. The patient’s task is simply to press a button in response to various signals and tasks, thereby measuring reaction time, omissions and errors. The TAP is economical to handle, valid, reliable, less prone to instructor and observer biases, and learning effects are [12]*.* The psychometric properties of the TAP are described as good [12]. Sensitivity for variation of test performance in the various TAP subtests has been demonstrated [13,14]).

Patients underwent three tasks. Alertness was measured by the reaction time to 40 visual stimuli (an upright cross), and by the reaction time to the same 40 visual stimuli cued acoustically. This test took about five minutes. Divided attention was measured by the number of missed correct reactions to each 20 different simultaneously applied visual and acoustical stimuli. The visual task is to react to the numbers 01 and 10, and not to react to quite similar lines. The acoustical task includes a regular sequence of high (2000Hz) and low (1000Hz) beeps (Di-Da-Di-Da etc.), and the patient has to detect an irregularity (e.g. Di-Di or Da-Da) in the sequence. This test took about 5.4 minutes. Selective attention was measured by the number of errors in a go/no-go task. The patient has to react to a visual stimulus (cross), but not to its alternative (inclined cross) shown in a random 1:1 relation. This test needs about two minutes.

To statistically evaluate the hypothesis that diabetics perform different from non-diabetics after CABG, ANOVA for repeated measurements with independent variables diabetes-status (diabetics vs. non-diabetics) and time point (one day before CABG vs. one week after CABG) was used. For detection of some further influences on changes in cognitive performance analysis of stepwise regression with the factors anxiety, depression, age, extracorporeal circulation time and aortic clamping time was conducted. According to the TAP test instructions [14], dependent variables were the medians of the individual reaction times in alertness without and with audio-cueing; the number of omissions in divided attention, and the number of errors in the go-nogo paradigm. To explore data in more detail, significant ANOVA-effects were further investigated by the Fisher Least Significant Difference (LSD) post-hoc test. All tests were performed with STATISTICA 8 (StatSoft Inc, Tulsa; Ok). A p-value < 0.05 was defined significant. To define a change within each subject as meaningful the “reliable change index” approach was used. We determined the critical value as ±1.96. All values above are meaningful.

## Results

The assumptions (homogeneity of variance; tested by the Levene test, and exclusion of a not normal distribution tested by Kolmogorov-Smirnov test) for ANOVA were met. Preoperatively, there was no difference detected in attentional performance between diabetics and non-diabetics. ANOVA revealed a highly significant interaction of test time point and diabetes status in alertness without audio warning (*F*(1, 28) = 7.0; *p* = 0.01; effect size η^2^ (*eta-squared*) = 0.2) and alertness with audio warning (*F*(1, 28) = 4.9; *p* = 0.03). LSD analysis confirmed that the slower postoperative reacting was restricted to only diabetic patients (LSD *p*=0.002 vs. *p*=0.9, LSD *p*=0.01 vs. *p*=0.8, respectively). Although performance in divided attention significantly declined postoperatively (*F*(1, 28) = 5.5; *p* = 0.03; effect size η^2^ = 0.15), diabetes scarcely missed significance (LSD *p*=0.06 vs. 0.2). Selective attention was not found to be affected. Results and descriptives are depicted in Table 2. Analysis of regression revealed that none of the evaluated factors have an influence on shift of reaction time.

**Table 2:** Test results of patients with and without a history of diabetes mellitus pre- and post-CABG

**TAP test**	**Diabetics (n=10)**		**Non-diabetics (n=20)**	
	**Pre-op**	**Post-op**	**Pre-op**	**Post-op**
alertness, without audio cueing; median reaction time (ms)	270.5 ± 52.7	356.4 ± 165.5	277.4 ± 66.7	278.0 ± 59.5
ANOVA interaction (diabetes-status x timepoint) p=0.01	post-hoc LSD p=0.002		post-hoc LSD p=0.9	
alertness, with audio warning; median reaction time (ms)	279.0 ± 52.7	333.6 ± 120.0	279.2 ± 66.8	275.7 ± 49.9
ANOVA interaction (diabetes-status x timepoint) p=0.03	post-hoc LSD p=0.01		post-hoc LSD p=0.8	
Divided attention, mean number of omissions	3.8 ± 1.8	7.0 ± 4.6	2.2 ± 2.3	3.9 ± 5.8
ANOVA main effect (timepoint) p=0.03; effect size η^2^ = 0.16	post-hoc LSD p=0.06		post-hoc LSD p=0.2	
selective attention, mean number of errors	0.7 ± 1.3	0.5 ± 0.7	0. 5 ± 0.8	1.1 ± 2.2
no significant ANOVA-effects				

In five of the ten (50%) diabetics the slowdown in alertness without audio warning was meaningful, whereas only one out of twenty (5%) of the non-diabetics showed a meaningful deterioration, and two (10%) reacted meaningfully faster after CABG. In alertness with audio warning, three (30%) diabetics had a meaningful slowdown. Four (20%) non diabetics reacted meaningfully faster and one (5%) reacted meaningfully slower.

## Discussion

The principal finding of our pilot study indicates that patients even with actually well controlled type 2 diabetes mellitus may be at risk for a more severe cognitive dysfunction after CABG in terms of newly acquired attentional deficits. Attention is the basis for a good performance in many tasks, best illustrated by the deficits seen in vascular dementia [6]. Usually, previous studies on postoperative abilities did assess many domains but nearly neglected attention [15]. Thus, patients with microvascular encephalopathy may be not correctly identified to be at risk for a more prominent postoperative brain dysfunction because the tests used were not adequate to detect the specific ability probably most severely affected.

It seems plausible that patients with a history of diabetes mellitus per se are at special risk for brain dysfunction undergoing surgery using extracorporeal circulation which affects brain perfusion. First, the presence of vascular risk factors accelerate a cognitive decline in terms of vascular dementia [16] which again is prominent in diabetes [5,17], especially in case of comorbid arterial hypertension [18] and aging [19]. Indeed, all of our patients – diabetics and non-diabetics – did also have a history of comorbid arterial hypertension. Second, diabetes includes the risk to develop cerebral microangiopathy, and it has been evaluated that half of the pre-CABG patients showed lacunar infarcts on CT corresponding to poorer performance [20].

Our findings do not preclude that the diabetic patients investigated here had also developed more severe postoperative dysfunction in other domains. However, we aimed on a methodological proof of principle and conclude that there should be more interest than until now in measurement of attention instead of using laborious test compositions with complicated definitions of what means a cognitive decline [21] when perioperatively investigating patients with vascular risk factors or prospectively evaluating the risk of postoperative delirium.

Though easy to apply, the test system used has been thoroughly evaluated in terms of psychometric properties, interrater stability, sensitivity for changes over time, repeated testing with very few training effects, and stability against disruption [7,12,14,15].

The small number of study patients may be perceived to limit the strength of our conclusion. However, this small number resulted from a straightforward selection of patients in terms of age, sex, medical history and vascular comorbidities, coronary as well as surgical characteristics, and exclusion of stroke patients either historically or perioperatively, together with an expected very high number of patient exclusions by postoperative complications, medications or intraoperative features. This depth of selection probably allows for an almost perfect comparison of patients with and without diabetes. Of course, a larger patient series together with application of multiple tests for other domains and MRI to visualize the severity of cerebral microangiopathy should follow this proof-of-principle study. With larger cohorts, also questions regarding the influence of diabetes duration, effects of the quality of diabetes management, or gender effects may be answered.

## Conclusion

Diabetics showed more severe post-CABG attentional deficits than well-matched non-diabetics probably reflecting the impact of diabetic brain microangiopathy. Testing of attention should be included in common test compositions to identify diabetic patients in special risk for postoperative delirium and poor performance.

This publication was funded by the German Research Foundation (DFG) and the University of Wuerzburg in the funding programme Open Access Publishing.

## Competing interests

The authors declare that they have no competing interests.

## Authors’ contributions

J-HK, WM made the conceptual design, performed statistics, interpreted data, wrote first draft of the manuscript. TT collected data and performed statistics. RL and JB interpreted data and revised the manuscript critically. All authors read and approved the final manuscript.
